# Case 3/2015 – 53-Year-Old Female with Cardiogenic Shock 12 Years after Surgical Correction of Endomyocardial Fibrosis

**DOI:** 10.5935/abc.20150119

**Published:** 2015-09

**Authors:** Michel Abi Kalansky Ponczek, Fernanda Seligmann Feitosa, Léa Maria Macruz Ferreira Demarchi

**Affiliations:** Instituto do Coração (InCor) – HC-FMUSP, São Paulo, SP – Brazil

**Keywords:** Choque Cardiogênico, Fibrose Endomiocárdica / cirurgia, Arritmias Cardíacas, Marca-Passo Artificial

A 53-year-old female, submitted to surgical correction of endomyocardial fibrosis (EMF) 12 years before, sought medical care with hypotension and bradycardia.

She complained of palpitations since the age of 29 years. Her symptoms aggravated 5 years later, with the appearance of dyspnea on moderate exertion. After 5 more years, the dyspnea intensified, being triggered on mild exertion and in the dorsal decubitus position. The patient was then referred to a hospital.

In 1994, the physical examination revealed irregular pulse, heart rate of 88 bpm, blood pressure of 104/80 mm Hg, and increased jugular venous pressure. The pulmonary exam was normal, and the heart auscultation showed arrhythmic heart sounds and mitral systolic murmur (+/4). The exam of the abdomen and limbs was within the normal range.

In February 1994, the electrocardiogram (ECG) showed atrial fibrillation, left bundle-branch block and left ventricular hypertrophy (Figure 1).

**Figure 1:**
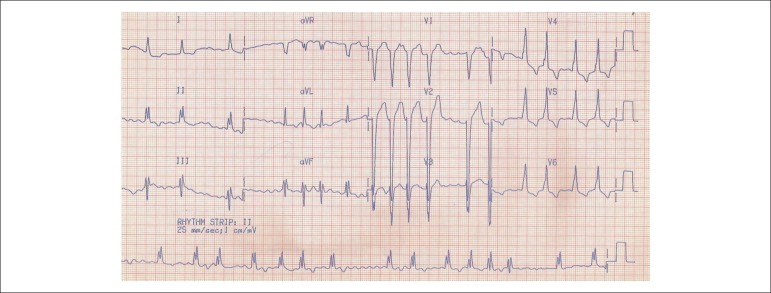
ECG: Atrial fibrillation, left bundle-branch block and left ventricular hypertrophy.

In 1996, on echocardiogram, the dimensions of the cardiac chambers were: left atrium, 57 mm; left ventricle, 51 mm (diastole) and 33 mm (systole). Obliteration of the apical region of both ventricles was observed, with suggestive signs of calcification. The morphological aspect was considered suggestive of EMF.

In 1996, a ventriculography showed obliteration of the apical region of left and right ventricles, in addition to mild mitral regurgitation. On coronary cineangiography, the circumflex branch of the left coronary artery originated from the right coronary artery. No obstruction of the coronary arteries was identified. The ventriculography findings were suggestive of EMF.

Surgical treatment was indicated.

In September 1996 the surgery was performed with incision in the apical region of the left ventricle and resection of a fibrous and calcified mass. Then, through the interatrial septum, annuloplasty with bovine pericardium was performed in the posterior portion of the mitral ring and in the anterior portion of the tricuspid ring.

The control ventriculography one month after surgery showed mild left ventricular hypokinesia and competent valves.

According to the patient, the dyspnea improved, but the palpitations remained.

She reported a transient episode of speech difficulty and right hemiparesis in 2002. The dyspnea reappeared, initially on moderate exertion and then on mild exertion.

A new echocardiographic assessment detected marked tricuspid regurgitation.

The medication was adjusted, with the prescription of the following drugs: warfarin, 5 mg; losartan, 50 mg; furosemide, 80 mg; digoxin, 0.25 mg; and diltiazem, 180 mg.

The symptoms improved, although episodes of aggravation occurred.

In 2005, a new hemodynamic study revealed hypertension of the right chambers. Left ventricular hypokinesia of moderate intensity in the inferior and apical walls, and of mild intensity in the anterior wall, was identified.

The pharmacological treatment was maintained, with adjustments according to symptoms. The patient was brought to the hospital after 24 hours of vertigo, malaise and dyspnea.

The physical examination on November 13, 2008, revealed pulse of 30 bpm, inaudible blood pressure, crepitant rales in pulmonary bases, bradycardia, liver palpable 7 cm from the costal margin, ascitis, and mild lower limb edema.

The ECG revealed atrial fibrillation, high atrioventricular block and ventricular rate of 30 bpm.

Chest X-ray showed enlarged cardiac area (+++/4+) and no signs of pulmonary congestion.

The laboratory findings on that occasion were as follows: potassium, 4.4 mEq/L; sodium, 134 mEq/L; urea, 100 mg/dL; creatinine, 1.45 mg/L (glomerular filtration rate, 40 mL/min/1.73m^2^); INR, 2.6; BNP, 250 pg/mL; hemoglobin, 12.6 g/dL; hematocrit, 40%; leukocytes/mm^3^, 7900 (78% neutrophils, 10% lymphocytes, and 12% monocytes); platelets/mm^3^, 183000; arterial lactate, 11 mg/dL; and blood digoxin level, 1.13 ng/mL.

Atropine and dopamine were administered and blood volume, replaced, but with no heart rate increase. Dobutamine was then administered, but unsuccessfully. A temporary transvenous external pacemaker was implanted, after which, blood pressure increased to 106/60 mmHg.

Permanent pacemaker was implanted on November, 19, 2008. The underlying disease posed technical difficulty, and a reserve electrode was implanted in the coronary sinus for an occasional capture loss of the right ventricular electrode.

The laboratory findings on that occasion were as follows: hemoglobin, 9 g/dL; leukocytes, 4700/mm^3^; platelets, 114000/mm^3^; urea, 47 mg/dL; creatinine, 0.76 mg/dL; potassium, 4.6 mEq/L; sodium, 133 mEq/L; blood glucose, 122 mg/dL; hemoglobin, 11.8 g/dl; hematocrit, 36%; and lactate; 7 mg/dL.

Oliguria and renal failure followed.

On December 4, 2008, the laboratory findings were as follows: creatinine, 2.4 mg/dL; urea, 105 mg/dL; potassium, 5.3 mEq/L; sodium, 130 mEq/L; hemoglobin, 8.8 g/dL; hematocrit, 30%; leukocytes, 6200/mm^3^ (neutrophils 86%, lymphocytes 4%, and monocytes 10%); platelets, 99000/mm^3^.

The patient had ventricular fibrillation and cardiac arrest, which did not respond to the resuscitation maneuvers, dying on December 5, 2008.

## Clinical aspects

At the age of 29 years, the patient developed palpitations, which, after 5 years, associated with dyspnea on exertion, which progressed slowly until the age of 39 years. In young patients, the major causes of heart failure (HF) are idiopathic dilated cardiomyopathy, myocarditis, Chagas disease, alcoholic cardiomyopathy, and, more rarely, ischemic heart disease. In addition, rarer causes of HF in patients of tropical and subtropical regions, such as EMF, are worthy of note.

When the patient first sought the hospital, she was diagnosed with EMF, which is an uncommon restrictive cardiomyopathy, accounting for 1.6% of the deaths due to cardiac disease in Brazil^1^. It is more common in the eastern and central regions of Africa. Several cases have been reported in South America and India, and sporadic ones have been reported worldwide. In some places, EMF has an endemic distribution.

Its etiology has not been completely clarified. Its pathogenesis is believed to involve several factors, such as environmental exposure, nutritional and immune abnormalities, in addition to genetic factors. One of the most accepted hypotheses is that of a more advanced spectrum of Loeffler's disease, caused by eosinophilic infiltration of the heart layers, with chronic inflammation and posterior fibrosis^2-4^. That pathogenic process favors thrombosis, usually in the ventricular apex.

The disease is characterized by deposition of fibrous tissue in the endocardium and, to a lesser extent, in the myocardium, usually affecting the apical region of the right or left ventricle, or both. A previous study has shown biventricular impairment in 50% of the cases, of the left ventricle in 30%, and of the right ventricle in 27%^2^. In addition, EMF usually affects the ventricular inlet, the papillary muscles and the valvular apparatus, mainly the mitral valve^3^.

In most patients, similarly to ours, the symptoms begin during adolescence, in the second or third decades of life. The initial symptoms relate to diastolic dysfunction, the systolic dysfunction being uncommon, except for the advanced stages of EMF^5^. Ascitis can occur in 50% of the patients, usually associated with important right ventricular fibrosis, worsening the prognosis^6^. Our patient had ascitis only in final stage of disease. Supraventricular arrhythmias can be observed in 60% of the patients, atrial fibrillation, as seen in our patient, being the most common^7^.

The diagnosis of EMF is established based on echocardiographic findings, which do not correlate with the severity of symptoms. The most common echocardiographic findings are changes in relaxation and compliance of the affected chamber, but usually without enlargement of the ventricular volumes. The atria are often enlarged and dysfunctional. Regurgitation of the atrioventricular valves is common. Pericardial effusion is usually present, and can be important.

Our patient's echocardiogram was compatible with the diagnosis of EMF, with marked enlargement of the left atrium (57 mm) and preservation of the ventricular diameters. Biventricular impairment with suggestive signs of calcification was observed.

In addition, our patient underwent ventriculography, which confirmed the biventricular involvement with mild mitral regurgitation. The hemodynamic study of patients with EMF usually reveals elevation of the end-diastolic pressure of the ventricle affected, restrictive pattern in the ventricular pressure recordings and obliteration of the ventricular apex^8,9^.

The prognosis of EMF depends mainly on its clinical presentation. The following factors are considered to worsen the prognosis: right ventricular impairment, ascitis, atrial fibrillation, mitral insufficiency, and increased atrial, end-diastolic ventricular and pulmonary pressures (> 40 mm Hg). The major factor determining the prognosis, however, is functional class (FC). A study has assessed survival according to the FC. Of the patients with FC I and II, 85% survive two years^8^, while less than 30% of those with FC IV survive two years^10^.

Thus, the treatment of EMF is based on symptoms. The recommendation for patients with few symptoms (FC I and II) is clinical treatment^11^, while that for more symptomatic ones [congestive HF (CHF) FC III and IV] is surgical treatment^12,13^. In the presence of worse prognostic factors, some experts recommend surgery already for FC II.

Clinical treatment is extrapolated from the clinical treatment for diastolic HF, being based on the following: diuretics to control blood volume; beta-blockers or calcium channel blockers to control heart rate in patients with AF or atrial tachycardia; and vitamin K antagonists for patients with intracardiac thrombi.

Our patient had indication for those drugs, including anticoagulation, because she already had had a thromboembolic event in 2002.

The surgical treatment consists in resecting the area of endocardial fibrosis and in correcting mitral or tricuspid regurgitation, when present, with valvular replacement or repair.

The initial surgical technique consists in replacing the impaired valves with prostheses. Later studies have shown that valvoplasty with native valve preservation related to lower morbidity and mortality.

The mortality related to classical surgery is extremely high, ranging from 20% to 30%^12,13^; thus, whenever possible, the native valve should be preserved.

Surgery determines an important improvement in symptoms and survival in symptomatic patients, and should always be performed in such patients.

In 1999, Moraes et al^14^ published a study, assessing 83 patients submitted to surgery for EMF, 45% of whom had CHF, FC I and II, and found a 55% survival in 17 years.

Recurrence of valvular regurgitation after surgery is not rare, and many patients might require a new surgery to fix the problem^14^.

Moraes et al^15^, assessing 107 patients undergoing surgery, have found a 4.5% incidence of fibrosis recurrence. The surgical mortality related to reoperation in such cases was extremely high, reaching 50%^15^.

Considering the high mortality associated with reoperation, the best treatment for that group remains uncertain. The heart transplantation team of the Instituto do Coração of the Hospital das Clínicas of FMUSP has reported the good outcome and survival of one patient with EMF previously submitted to surgery, but with recurrence of disease and important symptoms of HF. The possibility that, for that group of patients, cardiac transplantation can be more beneficial than reoperation has been raised^16^.

In 1996, our patient underwent surgery for the resection of the endomyocardial fibrosis, as well as mitral and tricuspid valvoplasty, because of important HF symptoms (FC III). Initially, her symptoms improved, which was confirmed on the ventriculography performed one month after surgery, showing competent valves and mild ventricular hypokinesia.

A few years after surgery, the HF symptoms recurred and a new echocardiogram showed important tricuspid regurgitation. A new hemodynamic study was performed in 2005, showing hypertension of the right chambers and moderate left ventricular hypokinesia, raising the suspicion of disease relapse. On the occasion, clinical treatment was chosen, but performing some tests, such as cardiac magnetic resonance, could have helped to establish the diagnosis of disease relapse.

The presence of hypertension of the right chambers could be related to HF itself, pulmonary thromboembolism, chronic obstructive pulmonary disease or other causes, requiring further investigation.

The patient's symptoms worsened and, on November 13, 2008, she sought the hospital with cardiogenic shock and important bradycardia, requiring transvenous pacemaker implantation. That bradycardia could have been caused by the use of medications to control heart rate, diltiazem and digoxin, because the fibrotic impairment of the cardiac conduction system is not common in EMF.

Despite all hemodynamic support and initial improvement, the patient's renal function worsened, raising the suspicion that her most likely cause of death was cardiogenic shock secondary to EMF relapse with valvular impairment.

The retrospective analysis of this case raised doubt whether a new surgery or heart transplantation in 2005, when the patient's CHF symptoms recurred, would have changed the disease course.

Further studies are required to help deciding on the best treatment for EMF relapse.

**(Michel Abi Kalansky Ponczek, MD, and Fernanda Seligmann Feitosa, MD)**

**Diagnostic hypothesis:** Endomyocardial fibrosis

(Michel Abi Kalansky Ponczek, MD, and Fernanda Seligmann Feitosa, MD)

## Postmortem examination

**Figure 2:**
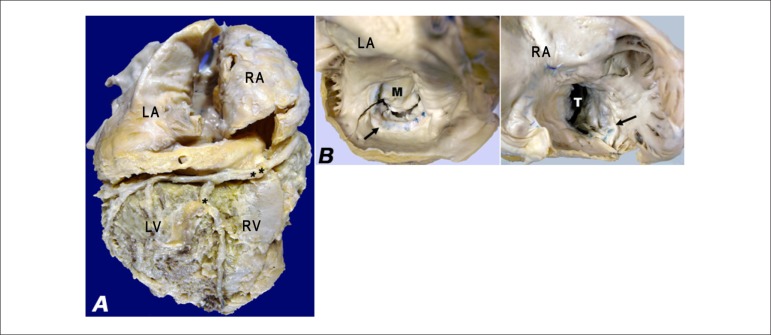
A - Heart, diaphragmatic surface: atrial volumes are proportionally greater than the ventricular ones. The right coronary artery is found in the atrioventricular sulcus (**), and the posterior interventricular branch of the right coronary artery, in the posterior ventricular sulcus (*). B - Left and right atria: bilateral endocardial thickening and surgical sutures of the old valvoplasty (arrows) in the posterior cusps (arrows) of the mitral (M) and tricuspid (T) valves.

**Figure 3:**
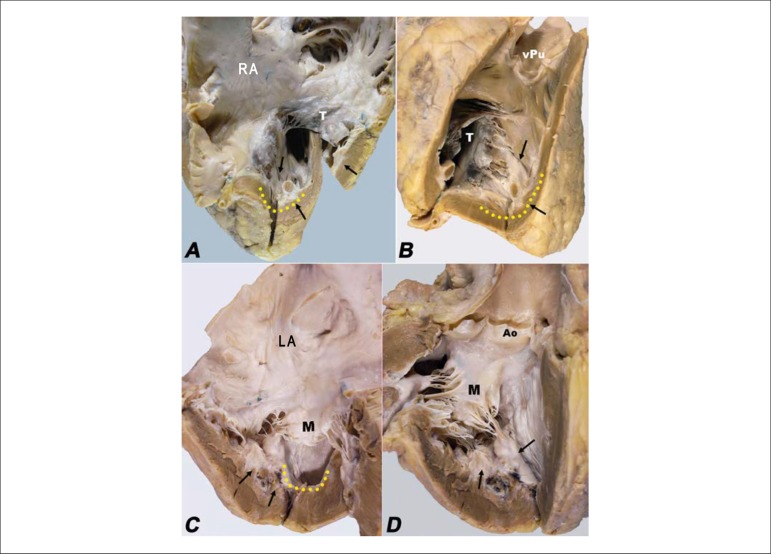
A and B - Right ventricular inlet and outflow tract: endocardial fibrosis affecting the ventricular inlet (arrows), with amputation of the ventricular apex (yellow dotted line), but sparing the outflow tract. C and D - Left ventricular inlet and outflow tract: endocardial fibrosis affecting the ventricular inlet and the papillary muscles of the mitral valve (arrows), amputation of the left ventricular apex (yellow dotted line) and extension to the ventricular outflow tract, but sparing the aortic valve (Ao).

**Figure 4:**
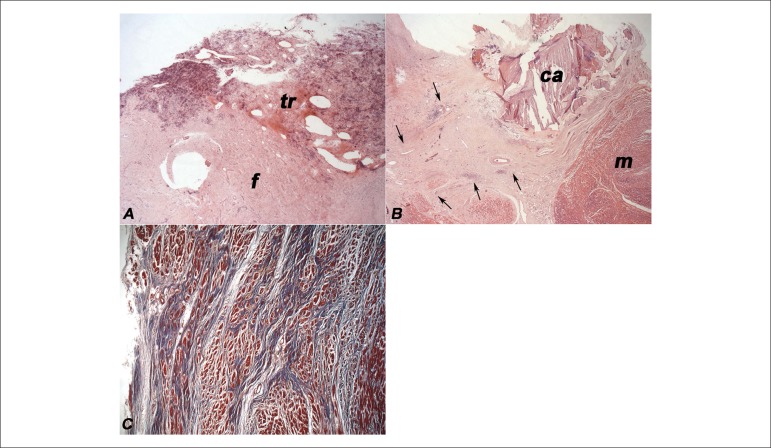
Endomyocardial photomicrograph: A - Organizing thrombus (tr) on the endocardium with intense fibrosis (f) (Hematoxylin-Eosin, 25x). B - Neovascularization and lymphoplasmacytic and mononuclear inflammatory infiltrate (between arrows) amid endomyocardial fibrosis, with calcified area (ca); m- myocardium (Hematoxylin Eosin, 25x). C - Irregular fibrosis bands (blue) surrounding cardiomyocytes (red) in the subendocardial region (Masson trichrome, 50x).

The heart weighed 652 g (normal: 200-300 g) and had multiple adherences between the visceral and parietal pericardium. Its volume was enlarged, especially because of the atria, whose volume was proportionally greater than that of the ventricles (Figure 2A). Opening of the heart revealed marked atrial dilatation and bilateral moderate endocardial thickening. The rings of the mitral and tricuspid valves showed surgical sutures of older valvoplasties, close to the insertion of the posterior cusps (Figure 2B). The right atrial roof also showed an old surgical suture. Endocardial thickening with areas of calcification was more intense in the ventricular inlet and apices, but partial in the left ventricular outflow tract, sparing the right ventricular outflow tract (Figure 3). The myocardial trabeculae were bound together due to endocardial thickening, which also involved the papillary muscles of the mitral and tricuspid valves, with marked reduction of the ventricular cavities and amputation of the ventricular tips (Figure 3). The chordae tendineae and cusps of the atrioventricular valves were not affected by endocardial thickening; however, there was moderate retraction of the free margins of the cusps and bilateral dilatation of the valvular ring. The right ventricular endocardium showed implanted electrodes of permanent biventricular cardiac pacemaker: one in the middle third of the interventricular septum and another in the anterolateral wall. There was thrombus on neither the pacemaker leads nor their implantation sites. On gross examination, no thrombi were found in the cardiac cavities, and the ventriculoarterial valves showed no abnormalities. The epicardial coronary arteries had no important obstructive changes. The microscopic examination revealed intense endocardial fibrosis and calcification, neovascularization areas and sparse lymphomononuclear inflammatory infiltrate, with expansion of irregular fibrous bands to the subjacent myocardium (Figure 4). Eosinophils were present in neither the endocardial inflammatory infiltrate nor other organs. The kidneys showed multiple and extensive healed cortical infarctions. In addition, morphological changes due to CHF, such as diffuse edema of the subcutaneous tissue, ascitis (1500 mL of yellow hyaline fluid) and chronic passive congestion in the lungs, liver and spleen, were observed. The cause of death was low cardiac output secondary to CHF, histologically represented by acute tubular necrosis and centrilobular necrosis of the liver.

**(Léa Maria Macruz Ferrreira Demarchi, MD)**

**Anatomopathological diagnoses:**

1) Biventricular endomyocardial fibrosis, operated on; 2) Congestive heart failure; 3) Low cardiac output.

**(Léa Maria Macruz Ferrreira Demarchi, MD)**

## Comments

The cardiac lesions revealed on the postmortem examination describe the morphology of biventricular EMF. The major characteristic of EMF is the focal or diffuse thick endocardial fibrosis, which affects mainly the inlet and apical region of one or both ventricles^17^. The myocardial trabeculae can be bound together due to endocardial thickening, which can involve the papillary muscle, chordae tendineae and the cusps of the atrioventricular valves, causing valvular insufficiency. Although our patient had already undergone mitral and tricuspid valvoplasty, such valves still had morphological changes of insufficiency, such as valvular ring dilatation and retraction of the free margin of the cusps. Microscopically, EMF is represented by dense and collagen endocardial fibrosis, with fragmented and irregularly arranged elastic fibers, which affect the subjacent myocardium, whose close regions have vascular neoformation and a variable amount of lymphoplasmacytic and mononuclear inflammatory infiltrate, with or without eosinophils^18^. Although not always present, eosinophils in endocardial lesions and peripheral eosinophilia, observed in different studies of individuals with EMF, have been reported as one of the probable causes of that disease^19^, whose etiopathogenesis remains to be clarified^20^. Several authors have proposed that the eosinophilic form, also known as Loeffler's endocarditis, and EMF without eosinophils represent different stages of the same disease, because the morphological endocardial changes in the chronic stage cannot be differentiated in the patients with and without eosinophilia^21,22^. This can be seen in our patient, who had eosinophils in neither the endocardial inflammatory infiltrate nor other organs. As EMF progresses, the formation and organization of endocardial thrombosis contribute to the obliteration and reduction of the ventricular cavity, and advanced cases frequently show calcification amid the fibrosis^18^. Usually, atrial dilatation results from the ventricular restriction caused by the endomyocardial fibrosis and the mitral and tricuspid valvular insufficiency, as well as from the pulmonary vascular changes secondary to passive pulmonary congestion, as reported in our patient. The healed renal infarctions were very likely caused by embolism from the previous thrombosis in the left ventricle. It is worth noting the post-surgery 12-year survival of our patient, considering the extension of the EMF lesions found on the postmortem examination. The survival likelihood of patients with EMF submitted to surgery is approximately 70% ten years after surgery^23,24^. Such lesions very likely represent disease relapse, but the possibility that not all the tissue affected by EMF had been removed in the previous surgery should be kept in mind.

**(Léa Maria Macruz Ferrreira Demarchi, MD)**

**Section editor:** Alfredo José Mansur (ajmansur@incor.usp.br)

**Associated editors:** Desidério Favarato (dclfavarato@incor.usp.br)

Vera Demarchi Aiello (anpvera@incor.usp.br)
